# Supplementary material for: Proteome Profiling of Rabies-Infected and Uninfected Dog Brain Tissues, Cerebrospinal Fluids and Serum Samples
Source: Proteomes. 2025 Dec 15;13(4):66. doi: 10.3390/proteomes13040066 (PMC12736986; doi:10.3390/proteomes13040066)
Supplement: Supplementary file 1 [file proteomes-13-00066-s001.zip › proteomes-3885518-supplementary.pdf]

Table S1. Characteristics of the 45 Proteins that Overlapped Between Serum and Cerebrospinal Fluid

S/N	Protein	Gene	Function	Dysregulation
1	Anion exchange protein	SLC4A1	Functions both as a structural protein and transporter that mediates electroneutral anion exchange across the cell membrane.	Increased abundance of anion exchange protein has been involved in human hepatocellular carcinoma [75].
2	Phosphatidylcholine transfer protein	PC-TP	Facilitates the transfer of phosphatidylcholine (PC) molecules between cellular membranes by binding and delivering them to target sites. It may also contribute to lipid and glucose metabolism, thereby influencing insulin sensitivity and brown adipose tissue thermogenesis.	Increase in abundance of PC metabolism is linked to diseases like fatty liver disease, atherosclerosis, and insulin resistance [76].
3	PDZ and LIM domain 1	PDLIM1	Cytoskeletal scaffold for assembling protein complexes. Supports synapse formation and maintenance for neuron communication.	Increased abundance in a variety of tumors and plays essential roles in tumor initiation and progression [34].
4	Isocitrate dehydrogenase [NADP]	IDH1	An enzyme that plays a crucial role in the citric acid cycle by catalyzing the oxidative decarboxylation of isocitrate to α-ketoglutarate.	Increase in abundance has been shown to enhance proliferation of glioma cells through aerobic glycolysis [77].
5	Insulin-like growth factor-binding protein complex acid labile subunit	IGFALS	A serum protein that binds insulin-like growth factors, increasing their half-life and vascular localization	An increase in abundance of the IGF axis can cause growth disorders, affect prenatal development (acromegaly), and promote cancer progression [78].
6	Myosin light chain 9	MYL9	It is a myosin complex component that regulates myosin and actin interactions, playing a key role in cell contraction, proliferation, invasion, and overall cellular dynamics	Increased abundance of MYL9 plays a vital role in immune infiltration, tumor invasion, and metastasis of different kinds of cancer [79].
7	Afamin	AFM	It transports vitamin E in body fluids, and across the blood-brain barrier.	Afamin levels are increased in metabolic syndrome and obesity, showing strong correlations with the syndrome's components [80].
8	Nucleoside diphosphate kinase A	NME1	Plays a crucial role in maintaining cellular nucleotide levels by catalyzing phosphorylating reactions between nucleoside	Increased abundance of nucleoside diphosphate kinases promotes neurite outgrowth, while substitution with inactive forms

			triphosphates (NTPs) and nucleoside diphosphates (NDPs)	suppresses nerve growth factor activity [81].
9	Thioredoxin-disulfide reductase	TXN RD2	It functions primarily by reducing oxidized thioredoxin, a protein that plays a vital role in various cellular processes, including antioxidant defense, protein disulfide reduction, and redox signaling.	Elevated abundance is linked to tumor development and resistance to cancer chemoradiotherapy [82].
10	26S proteasome non-ATPase regulatory subunit 2	PSM D2	Essential for maintaining protein homeostasis by eliminating misfolded or damaged proteins that could disrupt cellular functions, as well as removing proteins that are no longer needed.	PSMD2 is highly abundant in breast cancer and altered immune cell infiltration [83].
11	Glycolipid transfer protein	GLTP	A soluble protein found in the cytoplasm and is thought to play a role in regulating glycolipid homeostasis and potentially in vesicular transport.	Increased abundance can result in glycolipid metabolic disorders and impaired vesicular trafficking, potentially contributing to obesity, diabetes, liver dysfunction, neuromuscular complications, and cardiovascular and kidney diseases [84].
12	Globin domain-containing protein	Globi ns	It is an oxygen- binding protein and is involved in transport.	Specifically, an imbalance in the production of globin chains, primarily leads to thalassemia [85].
13	Inositol oxygenase	MIO X	An enzyme that catalyzes the oxidative cleavage of myo-inositol to d-glucuronic acid, a key step in myo-inositol catabolism, with the product further metabolized to xylitol.	Increased abuncance, leading to altered inositol levels, is associated with diabetes, cancer, neurological disorders, and reproductive abnormalities [86].
14	Histone H4	H4C1 6	Histones are essential for regulating transcription, facilitating DNA repair and replication, and maintaining chromosomal stability.	Increase in abundance of histone H4 can affect DNA repair mechanisms and chromatin structure, potentially contributing to diseases like cancer [87].
15	26S proteasome regulatory subunit 10B	PSM C6	A subunit of the 26S proteasome, a crucial protein degradation complex. Ubiquitinated proteins are recognized, unfolded, and degraded by the proteasome.	As part of the proteasome, it can degrade proteins involved in activating the IFN pathway [33].
16	Collagen type III alpha 1 chain	COL3 A1	It provides structural support, elasticity, and aids in blood clotting and wound healing.	Increased abundance causes vascular Ehlers-Danlos syndrome, leading to weakened connective

				tissues and increased risk of vessel, gastrointestinal, and uterine rupture [88].
17	Carbonic anhydrase 2	CA2	Carbonic anhydrase catalyzes the conversion of carbon dioxide to bicarbonate and a proton, supporting CO_2 transport, pH regulation, and ion transport.	Increased abundance can disrupt acid-base balance, impair bone metabolism, and contribute to neurological disorders [89].
18	Globin A2	GLN A2	Hemoglobin A2 is a minor hemoglobin component with limited physiological function but serves as a key diagnostic marker for beta-thalassemia trait.	Reduced HbA_2 levels have been seen in iron deficiency, Hb Constant Spring trait, homozygous Hb Constant Spring [90].
19	Apolipoprotein C-IV	APO C4	Plays a role in lipid metabolism, particularly related to triglyceride transport and clearance.	Increase in abundance of the gene may influence circulating lipid levels and may be associated with coronary artery disease risk[35].
20	Delta-aminolevulinic acid dehydratase	ALA D	Catalyzes an early step in tetrapyrrole biosynthesis by binding two molecules of 5-aminolevulinate at distinct sites and condensing them to form porphobilinogen.	Increased abundance impairs heme synthesis, leading to ALAD deficiency porphyria, a rare genetic disorder characterized by toxic accumulation of aminolevulinic acid [91].
21	Small ribosomal subunit protein uS8	RPS1 5A	A ribosomal protein that binds directly to rRNA, organizing the small subunit's platform region, and participates in pre-rRNA processing and modification during ribosome biogenesis.	Increased abundance is primarily associated with Diamond-Blackfan anaemia [92].
22	Hyaluronan and proteoglycan link protein 1	HAP LN1	It stabilizes proteoglycan–hyaluronic acid aggregates in the extracellular cartilage matrix.	HAPLN1 abundance is increased during the differentiation of human aortic smooth muscle cells into a contractile state but downregulated during Platelet-derived growth factor-BB-induced. dedifferentiation [93].
23	26S proteasome non-ATPase regulatory subunit 3	PSM D3	It is involved in the ATP-dependent degradation of ubiquitinated proteins. participates in numerous cellular processes, including cell cycle progression, apoptosis, or DNA damage repair.	Analysis revealed that PSMD3 is highly abundant in multiple myeloma (MM) patients, with elevated levels significantly associated with poor patient survival [37].
24	Superoxide dismutase	sod2	Removes toxic superoxide radicals generated within cells,	Increased abundance leads to oxidative stress and inflammation;

			thereby protecting against oxidative damage and maintaining cellular homeostasis.	it has also been involved in diabetes mellitus [94].
25	Protein 4.1	EPB41	It is a cytoskeletal protein that stabilizes spectrin–actin interactions of erythrocytes, thereby regulating membrane mechanical stability and deformability.	Increased abundance has been implicated in cancer, with numerous studies demonstrating its potential as a diagnostic and prognostic biomarker for tumors [95].
26	IF rod domain-containing protein	No gene name yet	It plays a role in maintaining cellular structure and mechanical integrity. They form the cytoskeleton, providing structural support and resilience to cells. They also play roles in cell shape, cell division, and cell signaling.	An increase in abundance of Intermediate filament (IF) may destabilize intermediate filament networks, potentially compromising the cell's capacity to withstand mechanical stress [96].
27	Queuine tRNA-ribosyltransferase catalytic subunit 1	QTRT1	QTRT1 specifically catalyzes the exchange of a guanine residue at position 34 of specific tRNAs with queuine, a modified adenine base.	Altered abundance can contribute to the pathogenesis of inflammatory bowel disease and related disorders [97].
28	Nucleoside diphosphate kinase A	NME1	This enzyme maintains nucleotide homeostasis, supporting DNA/RNA synthesis, energy metabolism, and signal transduction.	Increase in abundance of nucleoside diphosphate kinases promotes neurite outgrowth and has been linked to lung tumor progression, while inactive forms suppress nerve growth factor activity [36].
29	Flavin reductase (NADPH)	BLVRB	This enzyme uses NADPH to reduce flavins (riboflavin, FMN, FAD) and supports processes such as electron transfer, iron reduction, and bioluminescence.	Disruptions in its activity impair redox balance, alter metabolic pathways, and compromise cellular responses to oxidative stress [98].
30	RNA transcription, translation and transport factor protein	RTRAF	It is crucial for gene expression, ensuring precise and efficient translation of genetic information into functional proteins.	Altered abundance causes neurodegenerative disorders, cancer, and developmental abnormalities [38].
31	Myoglobin	GNLG	Its primary function is to store oxygen and facilitate its diffusion within muscle tissues, while also protecting cells from reactive oxygen	Excessive release of myoglobin during muscle breakdown contributes to rhabdomyolysis, which can cause acute kidney injury and systemic complications [99].

			species through its pseudoperoxidase activity.	
32	Triggering receptor expressed on myeloid cells 2	TREM2	The gene encodes a myeloid cell receptor vital for immune regulation, skeletal and neural development, and microglial functions such as inflammation, phagocytosis, and survival.	It has been implicated in neurodegenerative disorders such as Nasu-Hakola disease and Alzheimer's disease, and may also contribute to Parkinson's disease and amyotrophic lateral sclerosis [39].
33	Actin interacting protein 3-like C-terminal domain-containing protein	KIAA1217	This domain regulates actin dynamics and structure, supporting essential processes like cell movement, adhesion, and division.	Altered abundance has contrasting effects: loss causes osteoporosis, excess promotes osteoarthritis and cancer, while increased abundance protects in neuromuscular disorders [100].
34	Glutamate--cysteine ligase	GCLC	It catalyzes the first step of glutathione biosynthesis, linking L-glutamate and L-cysteine in an ATP-dependent reaction to form gamma-glutamylcysteine.	Reduced abundance has been linked to development of oxidative stress and schizophrenia [42].
35	Ig-like domain-containing protein	LOC102724971	Their primary role is molecular recognition and binding, supporting key processes such as cell-cell interactions, adhesion, and immune responses.	Viruses exploit Ig-like domain proteins to evade host immunity by suppressing or inhibiting immune responses e.g., SARS-CoV-2 [40].
36	Peroxiredoxin-2	PRDX2	Detoxifies peroxides and regulates hydrogen peroxide–mediated signaling, influencing pathways such as growth factor and TNF-α signaling.	Increased abundance of PRDX2 has been linked to cancers like colon, cervical, breast, and prostate cancers, as well as inflammatory and neurological diseases [101].
37	Nucleoside diphosphate kinase	Ndk	Ndk plays diverse roles in cellular processes, including protein phosphorylation, gene transcription regulation, DNA repair, tumor metastasis, cell proliferation, differentiation, and development.	Altered abundance can disrupt nucleotide and energy metabolism, alter cell signaling, and contribute to diseases such as cancer and bacterial virulence [102].
38	Phosphatidylinositol-glycan-specific phospholipase D	GPLD1	This protein hydrolyzes the inositol phosphate bond of GPI-anchored proteins, releasing them from the cell membrane.	Reduced abundance of GPI-PLD protein may be involved in prion propagation in the brains of prion diseases [103].
39	PITH domain-containing protein 1	PITHD1	Promotes megakaryocyte differentiation by up-regulating Runt-related transcription factor 1 expression.	Altered abundance is linked to male infertility, characterized by abnormal sperm morphology and reduced motility [104].

40	Vacuolar protein sorting-associated protein VTA1 homolog	VTA1	It plays a key role in the endosomal multivesicular body pathway, where it mediates the sorting of membrane proteins destined for degradation.	Altered abundance is linked to malignant choroidal melanoma and neurodegenerative conditions such as frontotemporal dementia and amyotrophic lateral sclerosis [41].
41	Globin domain-containing protein	Globins	It is an oxygen-binding and is involved in transport.	Specifically, an imbalance in the production of globin chains, primarily leads to thalassemia [105].
42	Immunoglobulin domain-containing protein	ICAM3	It plays a role in cell-cell recognition, cell-surface receptors, muscle structure and the immune system.	Dysregulation causes autoimmune diseases, chronic infections, allergies, certain cancers such as lymphoma and multiple myeloma, and primary immunodeficiency diseases [106].
43	Isocitrate dehydrogenase 2 [NADP]	IDH2	An enzyme that plays a crucial role in the citric acid cycle by catalyzing the oxidative decarboxylation of isocitrate to α-ketoglutarate.	Increase in abundance has been shown to enhance proliferation of glioma cells through aerobic glycolysis [77].
44	Lipopolysaccharide-binding protein	LBP	Plays a role in the innate immune response by acting as an affinity enhancer for CD14, facilitating its association with lipopolysaccharide.	It induces signaling that contributes to the proinflammatory milieu in human obesity, promotes adipose dysfunction and defines the adipocyte death size [107].
45	Carbonic anhydrase	CA1	Catalyzes the reversible hydration of carbon dioxide. Can hydrate cyanamide to urea.	CA1 altered abundance is linked to inflammation, metabolic disorders, depression, cancer, and schistosomiasis [108].
